# Comparison of Whole Blood RNA Preservation Tubes and Novel Generation RNA Extraction Kits for Analysis of mRNA and MiRNA Profiles

**DOI:** 10.1371/journal.pone.0113298

**Published:** 2014-12-03

**Authors:** Madlen Häntzsch, Alexander Tolios, Frank Beutner, Dorothea Nagel, Joachim Thiery, Daniel Teupser, Lesca M. Holdt

**Affiliations:** 1 LIFE – Leipzig Research Center for Civilization Diseases, University Leipzig, Leipzig, Germany; 2 Institute of Laboratory Medicine, Clinical Chemistry and Molecular Diagnostics, University Hospital Leipzig, Leipzig, Germany; 3 Institute of Laboratory Medicine, Ludwig-Maximilians-University Munich, Munich, Germany; 4 Department of Cardiology, Heart Center, University Leipzig, Leipzig, Germany; Philipps University, Germany

## Abstract

**Background:**

Whole blood expression profiling is frequently performed using PAXgene (Qiagen) or Tempus (Life Technologies) tubes. Here, we compare 6 novel generation RNA isolation protocols with respect to RNA quantity, quality and recovery of mRNA and miRNA.

**Methods:**

3 PAXgene and 3 Tempus Tubes were collected from participants of the LIFE study with (n = 12) and without (n = 35) acute myocardial infarction (AMI). RNA was extracted with 4 manual protocols from Qiagen (PAXgene Blood miRNA Kit), Life Technologies (MagMAX for Stabilized Blood Tubes RNA Isolation Kit), and Norgen Biotek (Norgen Preserved Blood RNA Purification Kit I and Kit II), and 2 (semi-)automated protocols on the QIAsymphony (Qiagen) and MagMAX Express-96 Magnetic Particle Processor (Life Technologies). RNA quantity and quality was determined. For biological validation, RNA from 12 representative probands, extracted with all 6 kits (n = 72), was reverse transcribed and mRNAs (*matrix metalloproteinase 9*, *arginase 1*) and miRNAs (miR133a, miR1), shown to be altered by AMI, were analyzed.

**Results:**

RNA yields were highest using the Norgen Kit I with Tempus Tubes and lowest using the Norgen Kit II with PAXgene. The disease status was the second major determinant of RNA yields (LIFE-AMI 11.2 vs. LIFE 6.7 µg, p<0.001) followed by the choice of blood collection tube. (Semi-)automation reduced overall RNA extraction time but did not generally reduce hands-on-time. RNA yields and quality were comparable between manual and automated extraction protocols. mRNA expression was not affected by collection tubes and RNA extraction kits but by RT/qPCR reagents with exception of the Norgen Kit II, which led to mRNA depletion. For miRNAs, expression differences related to collection tubes (miR30b), RNA isolation (Norgen Kit II), and RT/qRT reagents (miR133a) were observed.

**Conclusion:**

We demonstrate that novel generation RNA isolation kits significantly differed with respect to RNA recovery and affected miRNA but not mRNA expression profiles.

## Introduction

Gene expression profiling in peripheral blood is frequently performed to identify susceptibility genes or biomarkers for human traits or diseases. Upon collection, RNA profiles change within minutes [1], [2]. Therefore, PAXgene (Qiagen) and Tempus (Life Technologies) Blood RNA Tubes, which allow instant preservation of RNA, are widely used. The suppliers of both types of tubes offer corresponding RNA extraction kits as well as reagents for downstream applications such as reverse transcription (RT) and quantitative real time (qRT)-PCR assays for quantification of target transcripts. Whereas older extraction kits were optimized for isolation of mRNA (PAXgene blood RNA Kit, Tempus Spin RNA Isolation Kit) [3], [4], more recent assays were designed to also efficiently extract miRNAs (PAXgene blood miRNA Kit, MagMAX for Stabilized Blood Tubes RNA Isolation).

In the meantime, additional suppliers have launched extraction kits for PAXgene and Tempus Blood RNA Tubes, which are also optimized for parallel mRNA and small RNA extraction (Norgen). Extraction methods vary depending on the manufacturer and are either based on silica-membranes (e.g. Qiagen, Norgen) or magnetic beads (e.g. Life Technologies) [5]. Whereas previous work has investigated quantity and quality of RNA isolated by kits using the same extraction principle from different preservation tubes [3], [6], [7], comparison of more recently available extraction kits, which are based on different extraction principles and allowing efficient parallel mRNA and miRNA extraction has not been performed, yet.

Besides miRNA/mRNA quality and quantity, the duration of extraction is critical and automated sample processing is warranted especially in larger studies. Qiagen and Life Technologies provide companion instruments to facilitate RNA extraction, allowing parallel processing of up to 96 samples. To date, however, a comparison of the duration of sample processing as well as required hands-on times using different manual and (semi-)automated extraction methods has not been performed.

After extraction, RNA expression may be analyzed by genome-wide approaches, either using array [8]–[10] or sequencing techniques [11]–[14]. Both are still cost-intensive and thus, quantitative RT-PCR (qRT-PCR) [15], [16], is frequently used for determination of target gene expression. qRT-PCR requires prior reverse transcription of RNA into cDNA. Both, mRNA and miRNA, can be reverse transcribed in parallel using Qiagen reagents with non-specific Oligo(dT) primers after polyadenylation of all RNAs, whereas mRNA and miRNA are independently reverse transcribed with Life Technologies reagents using non-specific primers for mRNA or stem loop-specific primers for each miRNA. To the best of our knowledge, studies comparing the effects of different blood collection tubes in combination with different downstream RT and qRT-PCR reagents are lacking.

Thus, the aim of the current study was (1) to compare different extraction systems (filter-based vs. magnetic beads; manual vs. automated) for PAXgene and Tempus Blood RNA Tubes with regard to the quantity and quality of RNA, (2) to test different extraction protocols for the duration of extraction, hands-on time and processing time per sample, and (3) to evaluate mRNA and miRNA expression depending on the collection tube, RNA extraction method, RT and qRT-PCR reagents that were used. Studies were performed in probands of the LIFE study, which is an cohort study of the population of Leipzig, and in the LIFE Heart substudy, which recruits patients with myocardial infarction (AMI) [17], [18]. For biological validation, expression of representative genes, which were previously shown to be altered by AMI, such as *matrix metalloproteinase 9* (*MMP9*) and *arginase 1* (*ARG1*) [19]–[21] and miRNAs miR133a and miR1 [15], [22]–[25], were investigated.

## Materials and Methods

### Ethics statement, blood collection and study design

**Figure 1:**
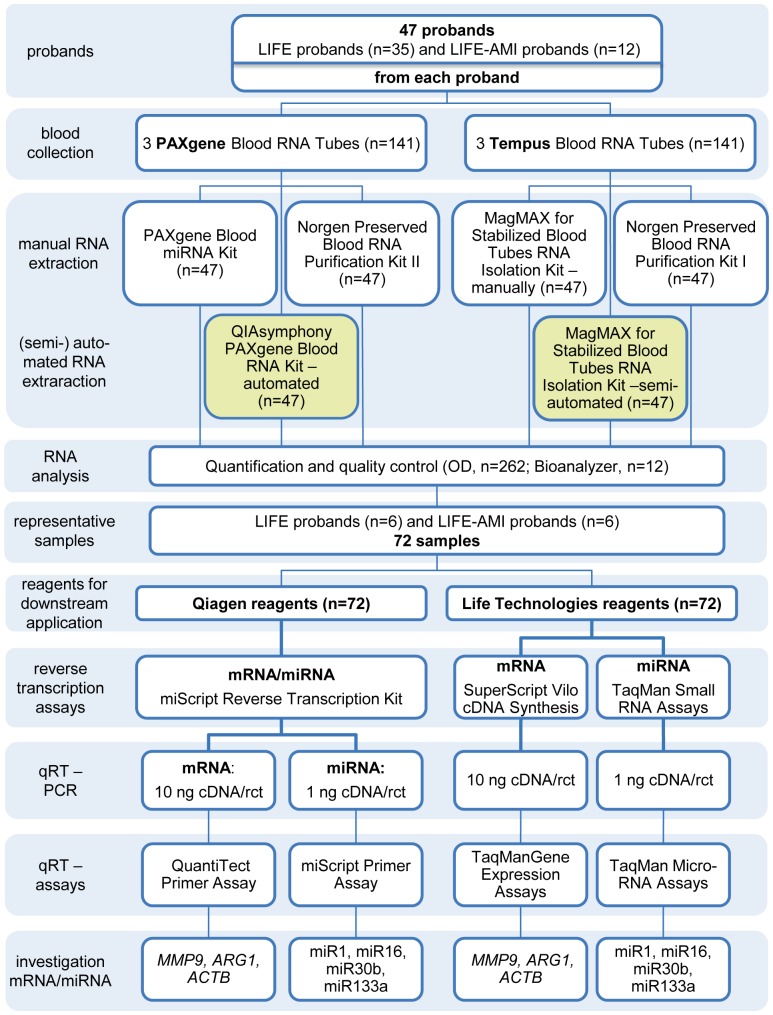
Study design. For comparison of extraction efficiency and RNA quality of whole blood stabilization tubes (PAXgene Blood RNA Tubes, Qiagen; Tempus Blood RNA Tubes, Life Technologies), blood was drawn from 47 probands of the LIFE and LIFE-AMI cohorts and RNA was isolated with 4 manual (white) and 2 (semi-)automated (light green) extraction kits. RNA quantity and quality was determined in 262 samples (20 extractions were lost due to handling errors). Samples from 12 probands were selected for RT and qRT-PCRs. Both steps were carried out with reagents from Qiagen or Life Technologies, respectively. Quantification of miRNA and mRNA expression was done using the ViiA7 Real-Time PCR System (Life Technologies). In Figure S1, Table S2 and Table S3, detailed information regarding the reaction setup for RT and qRT-PCR experiments is given. rct = reaction.

Studies were approved by the local ethics committee of the University of Leipzig (Reg. No 263-2009-14122009 and Reg. No 276-2005, respectively). Research participants gave informed written consent. Whole blood samples were collected from 35 probands of the LIFE study and 12 probands with acute myocardial infarction (AMI) from the LIFE Heart study [17] (Figure 1). Three PAXgene Blood RNA Tubes (Qiagen) and three Tempus Blood RNA Tubes (Life Technologies) were collected per proband. After blood collection, tubes were kept for 2 h at RT followed by incubation at -20°C for 24 h. Thereafter, tubes were transferred to -80°C for at least 7 days prior to processing.

### RNA isolation, quantification and quality control

6 RNA extraction protocols using 4 different RNA isolation kits were compared (Table 1). Four kits were used for manual RNA extraction: (1) PAXgene Blood miRNA Kit (Qiagen), (2) MagMAX for Stabilized Blood Tubes RNA Isolation Kit (Life Technologies), (3) Norgen Preserved Blood RNA Purification Kit I (Norgen I Kit), and (4) Norgen Preserved Blood RNA Purification Kit II (Norgen II Kit) from Norgen Biotek. Two kits were utilized for (semi-)automated RNA extraction: (5) QIAsymphony PAXgene Blood RNA Kit for fully automated RNA isolation on the QIAsymphony (Qiagen) and (6) MagMAX for Stabilized Blood Tubes RNA Isolation Kit for semi-automated isolation on the MagMAX Express-96 Magnetic Particle Processor (Life Technologies). The automated RNA extraction on the QIAsymphony was performed at the QIAGEN GmbH R&D Department in Hilden, Germany; all other extractions were done at the Institute of Laboratory Medicine, Clinical Chemistry and Molecular Diagnostics at the University of Leipzig, Germany. For each manual extraction, preservation tubes were thawed in batches of 24 PAXgene or 16 Tempus Blood RNA Tubes and processing was done according to manufacturers’ instructions including DNAse digestion. For the Norgen I Kit, DNAse treatment was performed with Norgen’s RNase-Free DNase I Kit, since it was not supplied with the extraction kit (Table 1). Before starting the extraction procedure, each blood tube was weighed for subsequent correlation with RNA yield (n = 282; Table S1). Purified RNA samples (n = 262) were quantified using the NanoDrop ND-1000 spectrophotometer (Fisher Scientific) and stored at −80°C. 20 extractions were lost due to handling errors. From two representative probands, RNA samples from all extractions (n = 6) were selected for quality testing using Agilent small RNA Kit run on the Bioanalyzer 2100 (Agilent Technologies) according to the manufacturer’s protocol.

**Table 1:** Summary of technical characteristics of applied RNA extraction methods from PAXgene and Tempus Blood RNA Tubes.

Blood stabilization	PAXgene Blood RNA Tubes			Tempus Blood RNA Tubes		
**Storage**	–80°C	–80°C	–80°C	–80°C	–80°C	–80°C
**Processing**	automated	manually	manually	automated	manually	manually
**Method**	QIAsymphony PAXgene Blood RNA Kit	PAXgene Blood miRNA Kit	Norgen Preserved Blood RNA Purification Kit II	MagMAX for Stabilized Blood Tubes RNA Isolation	MagMAX for Stabilized Blood Tubes RNA Isolation	Norgen Preserved Blood RNA Purification Kit I
**Principle isolation**	magnetic beads	spin columns	spin columns	magnetic beads	magnetic beads	spin columns
**Thawing**	2 h RT	2 h RT	2 h RT	30 min on ice	30 min on ice	30 min on ice
**Processing time/batch** a	145’/24 samples	205’/24 samples	152’/24 samples	139’/16 samples	162’/16 samples	152’/16 samples
**Av. total time/sample** b	6′ 30″	8′ 30″	6′ 30″	8′ 30″	10′	8′ 30″
**Centrifugation and** **vortexing time** a	15′	46′	43′	51′	55′	79′
**Incubation time** a	5′	30′	17′	2′	25′	17′
**Hands-on time** a	20′	129’	92′	69′	82′	56′
**Processing time on instrument** a	114’	–	–	30′	–	–
**Format on instrument**	24 batches (up to 96)	–	–	up to 96 samples	–	–
**DNAse digestion implemented in Kit**	yes	yes	yes	yes	yes	no^c^
**Elution volume**	200 µl	40 µl	50 µl	80 µl	80 µl	50 µl
**Required material/not supplied in Kit (additional to ethanol and isopropanol)**	8-rod covers, sample prep cartridges, RNAse-free syringe	–	RNAse-free micocentrifuge tubes	50 ml conical tubes; RNAse-free micocentrifuge tubes	50 ml conical tubes; RNAse-free micocentrifuge tubes	50 ml conical tubes; for on-column DNA removal: Norgen’s Rnase-Free DNase Kit I
**Required equipment**	QIAsymphony, thermomixer, swing bucket centrifuge (5000×g); vortexer	thermomixer, swing bucket centrifuge (5000×g); benchtop microcentrifuge, vortexer	thermomixer, swing bucket centrifuge, benchtop microcentrifuge, vortexer	MagMAX Express-96, magnetic stand, swing bucket centrifuge for 50 ml conical tubes (5000×g; 4°C), orbital shaker	magnetic stand, swing bucket centrifuge for 50 ml conical tubes (5000×g; 4°C), orbital shaker	thermomixer, swing bucket centrifuge for 50 ml conical tubes (5000×g; 4°C), benchtop microcentrifuge

a The time for centrifugation/vortexing, incubation steps and hands-on time was determined.

b During centrifugation, time was used for preparation of next steps including labeling. Thus, total duration might be different from sum of single steps. Av. = Average.

c DNase digestion was performed with RNase-Free DNase I Kit from Norgen Biotek.

### Reverse transcription (RT)

72 RNA samples, which were isolated with 6 RNA extraction protocols from 12 representative probands (6 LIFE and 6 LIFE-AMI probands), were selected for reverse transcription (RT) with Qiagen and Life Technologies reagents, respectively (Figure S1, Table S1, Table S2, Table S3). RT of mRNA and miRNA was performed in a single reaction using the miScript Reverse Transcription Kit from Qiagen (n = 72) with the miScript RT buffer supplied with the kit. Distinct assays from Life Technologies were necessary for RT of mRNA (Superscript VILO cDNA synthesis Kit; n = 72) and each miRNA using specific stem-loop-primers (TaqMan MicroRNA RT Kit, catalog number 4366596; n = 72 per miRNA). RT was performed with 450 ng total RNA using the Qiagen miScript Reverse Transcription Kit (for mRNA and miRNA) and the Life Technologies Superscript VILO cDNA synthesis Kit (for mRNA). The amount of RNA was defined based on the maximal RNA input volume of 14 µl for the Superscript VILO cDNA synthesis Kit from Life Technologies (Table S2, Table S3) and the lowest RNA concentration of 34.89 ng/µl (sample LIFE25, isolated with QIAsymphony, Table S1). 10 ng RNA was used for the TaqMan MicroRNA RT Kit for each investigated miRNA (Figure S1). All RT reactions were performed according to the manufacturers’ instructions using an ABI GenAmp PCR System 9700 (Applied Biosystems, Table S2, Table S3). cDNA samples were stored at −20°C until further use.

### Quantitative real time PCR (qRT-PCR)

For mRNA analyses, samples were diluted with RNase free water to a final amount of 10 ng template cDNA/sample in a volume of 2.5 µl. For miRNA quantification, 1 ng cDNA/sample/2.5 µl input were used (Table S2, Table S3). qRT-PCR quantification of mRNA and miRNA expression was performed using assays and accompanying reagents from either Qiagen or Life Technologies. Qiagen reagents: mRNA analyses were performed using Quantitect Primer Assays for *beta actin* (*ACTB*; QT01680476), *matrix metalloproteinase 9* (*MMP9*; QT00040040) and *arginase 1* (*ARG1*; QT00068446). miRNA quantification was performed with miScript Primer Assays for miR1 (MS00008358), miR16 (MS00031493), miR30b (MS00003276) and miR133a (MS00031423). Assays were run with the miScript SYBR Green PCR Kit for quantitative PCR (for further information see Figure S1 and Table S2). Life Technologies reagents: TaqMan Gene Expression Assays for *ACTB* (HS 99999903) *MMP9* (HS 00234579) and *ARG1* (HS 00968979) and TaqMan MicroRNA Assays for miR1 (ID 002222), miR16 (ID 000291), miR30b (ID 000602) and miR133a (ID 002246) were used in combination with the TaqMan Fast Advanced Mastermix. Reaction setups and manufacturers’ instructions are summarized in Figure S1, Table S2, Table S3. Measurements were performed in quadruplicates (384-well plates) in a final volume of 12.5 µl on the ViiA7 Real-Time PCR System (Life Technologies) according to the manufacturers’ instructions.

### Statistical analysis

Statistical analysis was done using SAS (Version 9.3, SAS Institute, Inc., Cary, NC, USA), GraphPad PRISM (Version 6.02, GraphPad Software, Inc., La Jolla, CA, USA), and Excel (Version 2010, Microsoft Corporation, Redmond, WA, USA). Normality of distribution was tested using the Kolmogorov-Smirnov test. For the calculation of RNA extraction efficiency, outliers deviating more than 2xSD from arithmetic mean were excluded from analysis. Comparison of two groups was done using Wilcoxon-Mann-Whitney test for non-normally distributed data, Welch’s t-test or Student’s t-test for normally distributed data with unequal or equal variances, respectively. Bonferroni correction for multiple testing was applied. The ΔCt and ΔΔCt method was calculated according to [26] using the mean Ct of LIFE probands as reference.

## Results

### Comparison of RNA isolation from PAXgene and Tempus Blood RNA Tubes with manual and (semi-) automated extraction kits

Two whole blood stabilizing systems, PAXgene blood RNA system (Qiagen) and Tempus RNA system (Life Technologies), were evaluated for total RNA yield, duration of RNA preparation and mRNA and miRNA preservation. To this end, three PAXgene and three Tempus Blood RNA Tubes were collected from each proband of the LIFE study with (n = 12) and without AMI (n = 35) [17] and RNA was extracted with either manual or (semi-)automated kits of the respective supplier and with kits from an alternative company (Norgen Biotek). The study design is shown in Figure 1. Table 1 provides information about the time for sample preparation, additional materials not supplied with the kits as well as required laboratory equipment.

A major difference between both types of stabilization tubes was the time recommended for thawing. According to the manufacturer’s instructions, PAXgene Blood RNA Tubes should be thawed for 2 h at room temperature whereas Tempus Blood RNA Tubes may be processed after 30 min thawing on ice. Upon thawing, 24 or 16 samples for PAXgene or Tempus Blood RNA Tubes, respectively, were processed in batches. The differences in numbers were due to the capacity of the available centrifuge (Heraeus Multifuge 3SR), which was limited to 16 conical tubes with a volume of 50 ml required for RNA isolation from Tempus Blood RNA Tubes with all investigated kits (Table 1).

Manual sample preparation of 24 PAXgene Blood RNA Tubes was faster using the Norgen Preserved Blood RNA Purification Kit II (152 min total with 92 min of hands-on time, compared to preparation with the PAXgene Blood miRNA Kit (205 min total with 129 min of hands-on time). Manual sample preparation for 16 Tempus Blood RNA Tubes was faster with the Norgen Preserved Blood RNA Purification Kit I (152 min total with 56 min of hands-on time), compared to the MagMAX Stabilized Blood Tubes RNA Isolation Kit (162 min total with 82 min of hands-on time).

Using the semi-automated MagMAX Express-96 Magnetic Particle Processor, RNA from 16 Tempus Blood RNA Tubes was isolated within 139 min, requiring 69 min of hands-on time. Using the fully-automated QIAsymphony, RNA from 24 PAXgene Blood RNA Tubes was isolated in 145 min requiring 20 min of hands-on time. Differences in hands-on times may largely be explained by the fact that the QIAsymphony is a fully automated instrument, in which thawed PAXgene Blood RNA Tubes can directly be loaded, whereas the MagMAX Express-96 Magnetic Particle Processor mainly supports washing and elution steps. To achieve the most economic use of reagents, it is recommended to work within batches of 24 samples on the QIAsymphony allowing parallel processing of up to 96 samples. In contrast, any number of samples (1 −96) can be processed with the MagMAX Express-96 Magnetic Particle Processor without loss of reagents.

**Figure 2:**
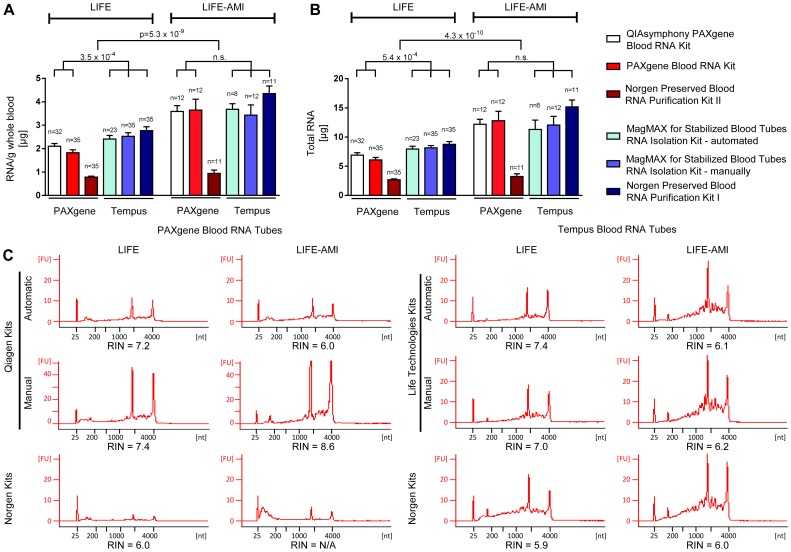
Quantity and quality of RNA preserved with PAXgene and Tempus Blood RNA Tubes. RNA quantity and quality were determined by OD and RNA integrity measurements. (A) RNA yield normalized to input whole blood volume (µg/g) and (B) absolute RNA recovery (µg). Significantly more RNA was recovered in samples from LIFE-AMI probands compared to LIFE probands (11.2 µg and 6.7 µg, respectively). In LIFE-AMI probands, the choice of collection tube did not affect RNA yields. In LIFE probands, higher RNA yields were recovered when using Tempus Blood RNA Tubes compared to PAXgene Blood RNA Tubes (8.3 µg and 6.5 µg, respectively). Outliers and missing values were omitted according to Table S1. Data are given as mean and SEM. (C) RNA samples extracted with six methods from representative LIFE and LIFE-AMI probands were analyzed on an Agilent Bioanalyzer. Please note the different scales in Figure 2C. RIN = RNA integrity number.

The average yields of all investigated RNA extraction methods are summarized in Figure 2, a full list of RNA yields, OD 260/280 and OD 260/230 ratios per sample is given in Table S1. Extraction of PAXgene Blood RNA Tubes with the Norgen Preserved Blood RNA Purification Kit II yielded significantly reduced RNA yields compared to all other PAXgene extraction methods for LIFE (*P*<10^–9^) and LIFE-AMI probands (*P*<10^–5^), respectively (Figure 2A,B). In contrast, extraction of Tempus Blood RNA Tubes with kits from Life Technologies and Norgen resulted in similar RNA yields (LIFE probands 8.5–9.3 µg; LIFE-AMI probands 12.7–15.2 µg).

In general, both the relative and total RNA yield from LIFE probands were approximately 30% higher for Tempus Blood RNA Tubes compared to PAXgene Blood RNA Tubes (Figure 2A) when using the manufacturers’ kits for RNA isolation. Relative and total RNA yields in LIFE-AMI probands were significantly higher than in LIFE probands and independent of the blood collection tube when using the manufacturer’s RNA extraction kits (Figure 2A,B). (Semi-)automated processing of samples revealed comparable RNA amounts as manual processing of the kits. RNA amounts recovered with all used RNA extraction kits were >500 ng per sample and thus sufficient for RNA sequencing using next-generation sequencing technology.

The quality of obtained RNA was investigated in representative samples using the Bioanalyzer 2100. Protein and organic compound contamination was calculated using the absorbance ratios at 230, 260 and 280 nm wavelength. On average, the 260/280 ratio was 2.04 in LIFE probands and 2.03 in LIFE-AMI probands, the 260/230 ratio was 1.01 in LIFE probands and 1.26 in LIFE-AMI probands. The electropherograms and RIN values for each extraction are illustrated in Figure 2C and revealed comparable results for all extraction methods (RIN values 5.9–8.6), although both Norgen kits were at the lower end of the scale (RIN 5.9–6.0).

### Biological validation of RT assays and absolute qRT-PCR quantification

For a more precise evaluation of obtained RNA, samples from 6 LIFE (mean RNA yield 7.4 µg) and 6 LIFE-AMI probands (mean RNA yield 10.4 µg) from all 6 extraction methods were used for reverse transcription and subsequent qRT-PCR (n = 72). All reactions were carried out with reagents from Qiagen or Life Technologies, respectively, and the same amount of RNA was used in all experiments. Schematics about all specifications and working steps are given in Figure S1, Table S2 and Table S3. Processing time, e.g. hands-on time and incubation steps, were comparable for RTs with Qiagen and Life Technologies reagents (Table S2). However, Qiagen reagents allowed simultaneous RT of both, mRNA and miRNA in only one reaction, whereas Life Technologies assays required 5 RT reactions (one for mRNA and 4 for miRNAs).

For biological validation, we quantified *MMP9* and *ARG1* expression, since both genes have been shown to be induced in AMI [19], [27]. *Beta actin* (*ACTB*) was selected as house-keeping gene. For miRNAs miR133a and miR1, an increased expression has been shown in AMI [15], [22], [23]. miR16 and miR30b were selected as house-keeping miRNA genes, since they are highly expressed in whole blood and expression was not altered in patients with AMI [15], .

**Figure 3:**
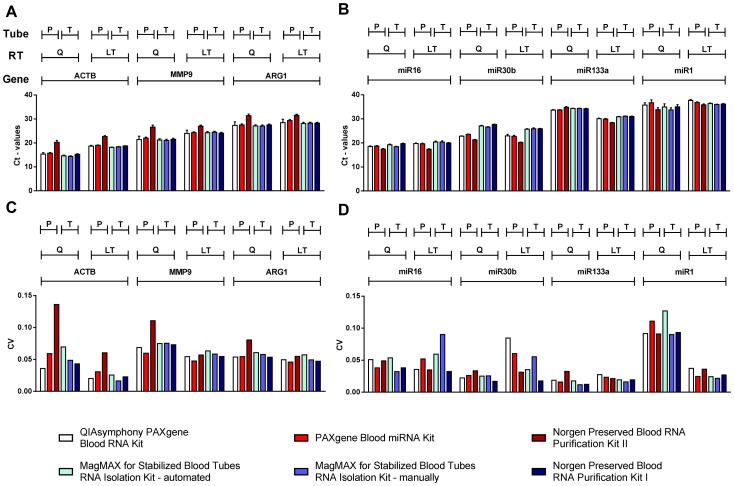
Analyses of absolute Ct-values and coefficients of variation of investigated mRNAs and miRNAs. RNA from 12 probands, extracted with 6 RNA isolation kits from two collection tubes (PAXgene [P] and Tempus Blood RNA Tubes [T]) (n = 72) was reverse transcribed and analyzed by qRT-PCRs with reagents from Qiagen (Q) and Life Technologies (LT). Results of qRT-PCRs showing mean Ct-values (error bars indicate SEM) for (A) mRNA and (B) miRNA transcripts. (C,D) corresponding mean coefficients of variation (CV).

In general, RNA extraction from PAXgene Blood RNA Tubes using the Norgen II Kit resulted in higher Ct-values for all quantified mRNA transcripts (Figure 3A) but lower Ct-values for all but one of the miRNA transcripts (Figure 3B) irrespective of the RT or qRT-PCR reagents that were used. This suggested an enrichment of miRNAs whereas mRNAs were less efficiently recovered by this kit.

For mRNA, we observed no major effects using different collection tubes or RNA isolation kits, whereas the use of RT and qRT-PCR reagents had a significant effect on mRNA expression profiles: Life Technologies reagents resulted in higher Ct-values, an effect more pronounced in higher expressed mRNAs (Figure 3A), which might be of relevance when using the ΔCt- and ΔΔCt-method for data normalization.

The miRNA expression data were less homogeneous. miRNA quantification was in part affected by the choice of collection tubes (e.g. mi30b, Figure 3B) and RT and qRT-PCR reagents (miR133a, Figure 3B) as well as the extraction kit (Norgen II Kit showed lower Ct-values in almost all analyzed samples). Notably, mRNA and miRNA expression was comparably high so that these effects could not be explained by lower abundance of investigated miRNAs (Figure 3A,B).

We next assessed assay robustness of mRNA/miRNA quantification by calculating the coefficient of variation (CV) of the Ct-values (Figure 3C,D). For all mRNA transcripts, the CVs were generally below 0.10. The exceptions were PAXgene Blood RNA samples isolated with the Norgen II Kit in combination with Qiagen reagents. Here, the CVs were 0.14 for *ACTB*, 0.11 for *MMP9*, and 0.08 for *ARG1*, respectively (Figure 3C). For miRNA quantification, all CVs were below 0.10 with the exception of the very low expressed miR1 when samples were reverse transcribed with Qiagen reagents (Figure 3D).

### Relative differences in mRNA and miRNA expression in LIFE and LIFE-AMI probands

**Figure 4:**
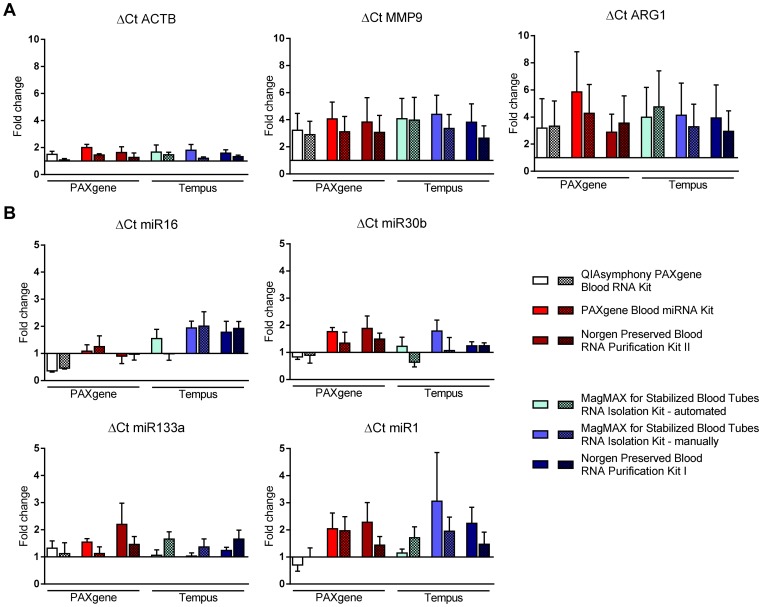
Biological validation of investigated mRNA and miRNA transcripts in LIFE-AMI and LIFE probands. ΔCt analysis of (A) *beta actin* (*ACTB*), *matrix metalloproteinase 9* (*MMP9*) and *arginase 1* (*ARG1*) expression and (B) miR16, miR30b, miR133a and miR1 expression in in LIFE-AMI probands compared to LIFE probands depending on the RNA extraction kit (n = 6) and RT/qRT-PCR reagents from Qiagen (non-shaded bars) and Life Technologies (shaded bars), respectively. Data are given as mean and SEM.

ΔCt analysis of *MMP9* and *ARG1* revealed a trend towards increased expression in LIFE-AMI compared to LIFE probands irrespective of the collection tube, RT or qRT-PCR reagents. Expression of house-keeping gene *ACTB* was not significantly altered between the two groups (Figure 4A). Consistent with this, ΔΔCt-analysis of *MMP9* and *ARG1* expression relative to *ACTB* showed a trend towards up-regulation of both genes in LIFE-AMI compared to LIFE probands (Figure S2), which is in agreement with published literature [19], [20], [29].

With respect to miRNA quantification, results were less consistent (Figure 4B). For miR16 and miR30b, which were initially chosen as house-keeping miRNAs, in part inverse effects were observed depending on the RNA collection tube, RT and qRT-PCR reagents (Figure 4B). Results for miR133a and miR1 were slightly more robust and showed a general trend towards increased expression in LIFE-AMI compared to LIFE probands (Figure 4B). ΔΔCt analysis of miR133a and miR1 relative to miR16 and miR30b revealed inconsistent results (Figure S2).

## Discussion

In the current study, we demonstrate significant differences in RNA quantity and quality after whole blood stabilization with PAXgene Blood RNA Tubes (Qiagen) and Tempus Blood RNA Tubes (Life Technologies) and RNA extraction with manual and (semi-)automated novel generation RNA isolation kits. Furthermore, we show that mRNA expression profiling was unaffected whereas miRNA expression levels were altered by the choice of collection tube, RNA extraction kits and RT/qRT-PCR reagents.

Previous studies investigated mRNA expression in PAXgene and Tempus Tubes using older generation RNA extraction kits [6], [30], [31], not specifically designed for extraction of miRNA. Here, we focused on novel generation kits, which allow parallel isolation of mRNA and miRNA. In addition to extraction kits, which were designed by the suppliers for use with the respective collection tubes (Qiagen, Life Technologies), we also evaluated RNA isolation kits from an alternate supplier (Norgen Biotek).

With respect to RNA yields, the Norgen Kit I in combination with Tempus Tubes revealed highest RNA yields, whereas the Norgen Kit II with PAXgene Tubes revealed the lowest RNA yields (60 −70% less), compared to all other isolation kits (Figure 2A,B). The disease status was the second major determinant of RNA yields followed by the choice of blood collection tube. Notably, RNA yields were comparable between manual and automated extraction protocols.

For biological validation, we used samples from apparently healthy individuals from a population-based cohort (LIFE) and a cohort of patients with acute myocardial infarction (LIFE-AMI) [17], where we found approximately 55% higher RNA yields compared to LIFE probands (Figure 2). This finding is in line with the observation that AMI was associated with an increased number of peripheral leucocytes [32], which are as a major source of whole blood RNA. Since leucocyte counts were not available, this correlation could, however, not be investigated in the current study. In addition, the choice of collection tubes had a significant impact on RNA yields. With Tempus Tubes, approximately 30% higher RNA yields were recovered compared to samples that were collected with PAXgene Tubes (Figure 2). Those results confirm findings from other groups [6], [30], [31], which found a 80% to 160% greater RNA yield using Tempus Blood RNA Tubes, respectively.

With respect to RNA quality, we found overall average RNA integrity numbers (RIN) of 5.9–8.6, average OD ratios 260/280>2.0, and 260/230>1.0 (Figure 2, Table S1), which was consistent with work from Duale et al [6]. Results were independent of the isolation principle of the kits (silica membranes-Qiagen vs. magnetic beads- Life Technologies). Notably, both kits from Norgen revealed RNA integrity numbers (RIN) of 5.9–6.0, which were at the lower end of the scale in comparison with the other investigated kits. These results highlight that the combination of collection tubes and isolation kits from Norgen might be less suitable in case of RNA quality-sensitive downstream analyses. For applications where RNA quality is not critical, these RNA isolation kits may be superior in RNA recovery and may reduce costs for RNA isolation.

We also evaluated the duration of sample preparation and compared manual and (semi-) automated extraction protocols. In general, automated extraction protocols were slightly faster than manual protocols. The fastest manual RNA extractions were those from Norgen Biotek for both, PAXgene and Tempus Tubes (152 min, each), which also required least hands-on-time compared to manual kits from Qiagen and Life Technologies (Table 1). The fastest automated extraction was the one using the MagMAX Express-96 Magnetic Particle Processor (Life Technologies, 139 min). As opposed to manual processing of the kits, automation with the QIAsymphony (Qiagen) or the MagMAX Express-96 Magnetic Particle Processor (Life Technologies) reduced the total extraction time by 30% and 14%, respectively, and led to a reduction of hands-on-time by 84% and 16%, respectively (Table 1). Notably, semi-automated processing of 16 Tempus Tubes required 23% longer hands-one-time compared to the fastest manual protocol for the same samples (69 min opposed to 56 min for the Norgen Preserved Blood RNA Kit I, Table 1). Thus, results of the current study highlight that counterintuitively, (semi-)automation did not lead to a reduction of hands-on-time in general.

We further evaluated potential impacts of the novel generation RNA isolation kits on mRNA and miRNA profiles. With exception of the Norgen Preserved Blood RNA Purification Kit II, we demonstrate that mRNA expression profiles of selected candidate genes were not affected by the type of collection tube and by different RNA isolation kits (Figure 3). *MMP9* and *ARG1* mRNA expression was investigated because these genes were shown to be induced in patients with AMI [19], [20], [29]. In the current study, we confirmed upregulation of both genes in LIFE-AMI (Figure 4), providing a biological validation of the kits used in this study.

In contrast, miRNA expression profiles were affected by the type of collection tubes (e.g. miR30b), RNA extraction kit (Norgen Preserved Blood RNA Purification Kit I, e.g. miR16, miR30b, miR133a, miR1), and RT/qRT-PCR reagents (e.g. miR133a) (Figure 3). miR1 and miR133a have been identified as markers of AMI [15], [23], however, the association of miRNA expression with disease status revealed inconsistent results in the current study (Figure 4). This might in part be explained by the small study size (LIFE-AMI (n = 6) vs. LIFE (n = 6)) but also by the aforementioned confounding effects of collection tubes and processing steps on miRNA quantification. Results from our study thus demonstrate that in principal, recovery of both, mRNA and miRNA, is achieved with novel generation RNA isolation kits but that standardization of sample processing including RT and qRT-PCR is essential, in particular when miRNA expression is investigated.

Taken together, our study shows significant differences in RNA recovery and quality using different RNA collection tubes in combination with manual and (semi-)automated novel generation RNA extraction kits. It demonstrates that mRNA expression was rather stable but miRNA levels were affected by the collection tubes and processing steps. In case of multi-center studies or replication studies, where samples might have been collected and processed according to different protocols, special attention should be paid to adjust for these potentially confounding factors.

## Supporting Information

Figure S1 **Extended information on reverse transcription (RT) and qRT-PCR study design.** RNA samples from 12 probands (6 LIFE probands, 6 LIFE-AMI probands) were selected for downstream application using reagents from Qiagen and Life Technologies. Analysis of mRNA and miRNA was carried out using the ViiA 7 (Life Technologies). The detailed experimental setup for RT and qRT-PCR is summarized in Table S2 and Table S3.
(TIF)

Figure S2 **RT and qRT-PCR results of selected mRNAs and miRNAs in dependency of RNA extraction kits.** Expression of (A) matrix metalloproteinase 9 (MMP9) and arginase 1 (ARG1) and (B) miR133a and miR1 in LIFE-AMI relative to LIFE probands. mRNA and miRNA was normalized to *beta-actin* (*ACTB*) or either to miR16 or miR30b, respectively. Fold changes and SEM were calculated according to [26]. Whereas differences in mRNA levels between kits were minor, major differences were found for miRNA levels. Absolute Ct-values are shown in Figure 3, non-normalized mean fold changes and SEM are illustrated in Figure 4.
(TIF)

Table S1 **Characteristics of the extracted RNA.** Total amount, concentration and OD values of RNA extracted from PAXgene Tubes (A) and Tempus Tubes (B) and blood weight for LIFE probands (LIFE1– LIFE35) and LIFE-AMI probands (LIFE-AMI1– LIFE-AMI12) were measured. Before starting the RNA extraction procedure, the RNA Blood tubes were weighted. Empty PAXgene Tubes and Tempus Tubes were used as tare weight. After each RNA extraction (using the six methods described in the manuscript), RNA yield was measured using Nanodrop. Samples were excluded (–) where total RNA amount was too low likely due to inaccurate handling. Furthermore, cells in green indicate RNA yield out of the 95% distribution. These values were omitted for the analyses in Figure 2. Samples marked in yellow were used for downstream analyses including reverse transcription and qRT-PCR (see Figure S1).
(XLSX)

Table S2 **Specifications of reverse transcription (RT) and qRT-PCR using reagents from Qiagen and Life Technologies.** Detailed information on the used volumes and dilutions is given in Figure S1 and Table S3. rct = reaction.
(XLSX)

Table S3 **Setup for RT and Real-time PCR.** Overview including all reaction setup for the processing of Reverse Transcription (RT) and quantitative real-time PCR using kits from Qiagen and Life Technologies. rct = reaction.
(XLSX)
