# Supplementary figures and images for: Comparison of Whole Blood RNA Preservation Tubes and Novel Generation RNA Extraction Kits for Analysis of mRNA and MiRNA Profiles
Source: PLoS One. 2014 Dec 3;9(12):e113298. doi: 10.1371/journal.pone.0113298 (PMC4254602; doi:10.1371/journal.pone.0113298)

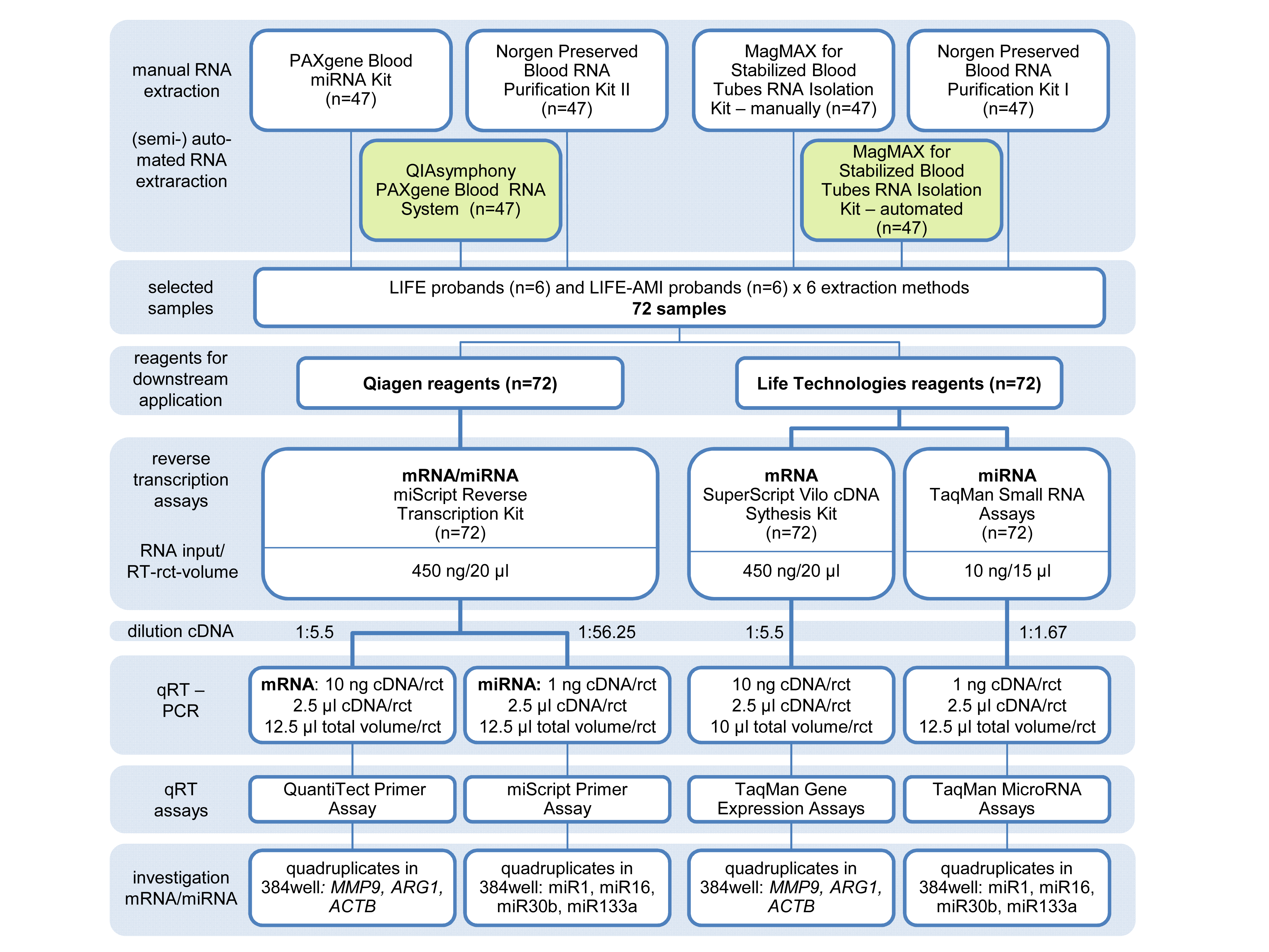

Supplement: Figure S1 — Extended information on reverse transcription (RT) and qRT-PCR study design. RNA samples from 12 probands (6 LIFE probands, 6 LIFE-AMI probands) were selected for downstream application using reagents from Qiagen and Life Technologies. Analysis of mRNA and miRNA was carried out using the ViiA 7 (Life Technologies). The detailed experimental setup for RT and qRT-PCR is summarized in Table S2 and Table S3. (TIF) [file pone.0113298.s001.tif]

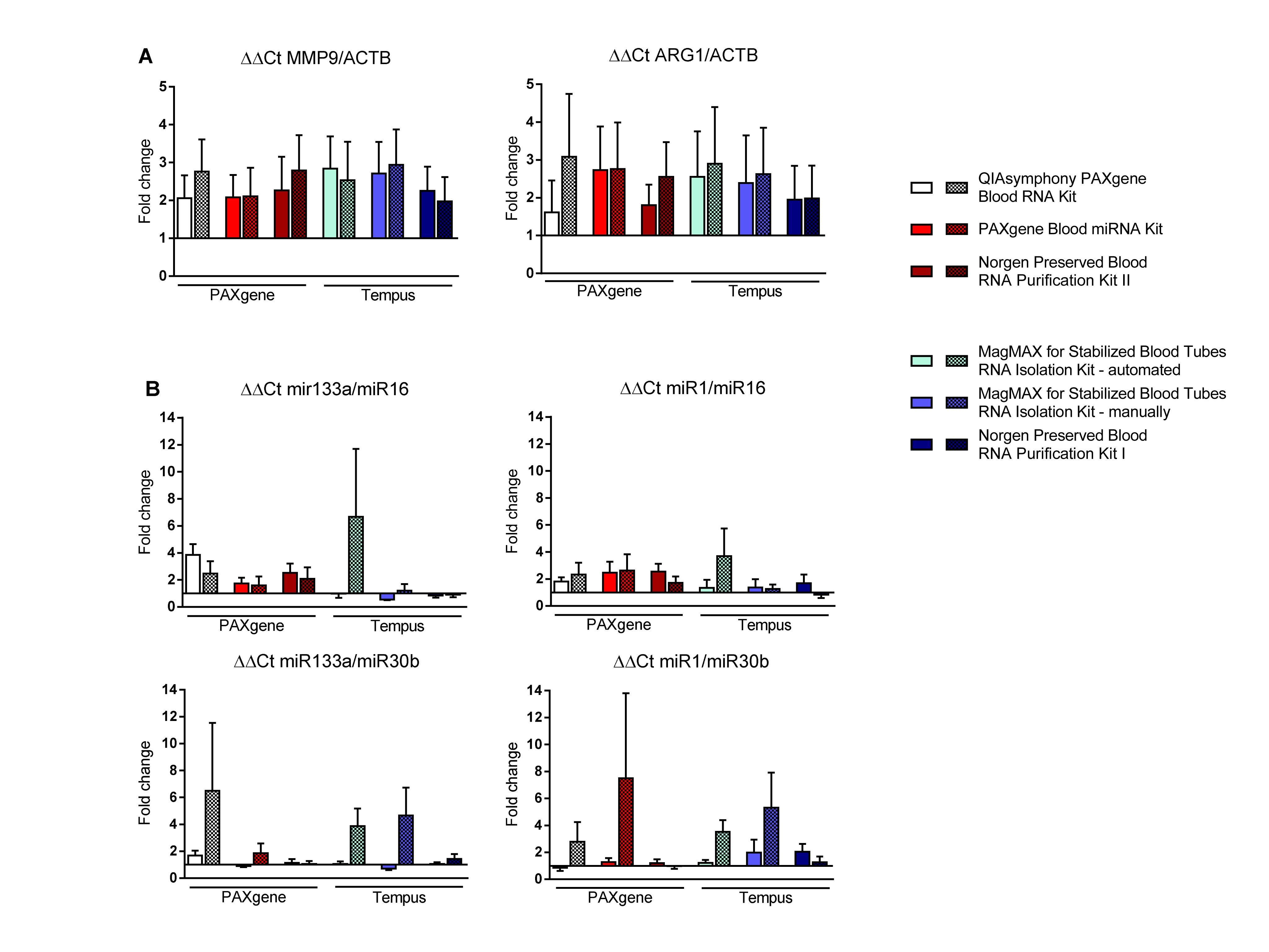

Supplement: Figure S2 — RT and qRT-PCR results of selected mRNAs and miRNAs in dependency of RNA extraction kits. Expression of (A) matrix metalloproteinase 9 (MMP9) and arginase 1 (ARG1) and (B) miR133a and miR1 in LIFE-AMI relative to LIFE probands. mRNA and miRNA was normalized to beta-actin (ACTB) or either to miR16 or miR30b, respectively. Fold changes and SEM were calculated according to [26]. Whereas differences in mRNA levels between kits were minor, major differences were found for miRNA levels. Absolute Ct-values are shown in Figure 3, non-normalized mean fold changes and SEM are illustrated in Figure 4. (TIF) [file pone.0113298.s002.tif]
